# A Galectin from the Kuruma Shrimp (*Marsupenaeus japonicus*) Functions as an Opsonin and Promotes Bacterial Clearance from Hemolymph

**DOI:** 10.1371/journal.pone.0091794

**Published:** 2014-03-11

**Authors:** Xiu-Zhen Shi, Lei Wang, Sen Xu, Xiao-Wen Zhang, Xiao-Fan Zhao, Gerardo Raul Vasta, Jin-Xing Wang

**Affiliations:** 1 MOE Key Laboratory of Plant Cell Engineering and Germplasm Innovation/Shandong Provincial Key Laboratory of Animal Cells and Developmental Biology, School of Life Sciences, Shandong University, Jinan, Shandong, China; 2 Department of Microbiology and Immunology, School of Medicine, University of Maryland Baltimore, and Institute of Marine and Environmental Technology, Baltimore, Maryland, United States of America; National Science and Technology Development Agency, Thailand

## Abstract

Galectins are a lectin family characterized by a conserved sequence motif in the carbohydrate recognition domain, which preferential binds to galactosyl moieties. However, few studies about the biological roles of galectins in invertebrates have been reported except for the galectin (*Cv*Gal1) from the eastern oyster *Crassostrea virginica*. Furthermore, galectins have been described in only a few crustacean species, and no functional studies have been reported so far. In this study, we identified and functionally characterized a galectin from the kuruma shrimp *Marsupenaeus japonicus,* which we designated *Mj*Gal. Upon *Vibrio anguillarum* challenge, expression of *Mj*Gal was up-regulated mostly in hemocytes and hepatopancreas, and the protein bound to both Gram-positive and Gram-negative bacteria through the recognition of lipoteichoic acid (LTA) or lipopolysaccharide (LPS), respectively. By also binding to the shrimp hemocyte surface, *Mj*Gal functions as an opsonin for microbial pathogens, promoting their phagocytosis. Further, as shown by RNA interference, *Mj*Gal participates in clearance of bacteria from circulation, and thereby contributes to the shrimp’s immune defense against infectious challenge. Elucidation of functional and mechanistic aspects of shrimp immunity will enable the development of novel strategies for intervention in infectious diseases currently affecting the shrimp farming industry worldwide.

## Introduction

Invertebrates lack the typical adaptive immune responses of vertebrates but are endowed with effective defense mechanisms against infectious challenge [1]. They comprise both cellular and humoral responses mediated by multiple soluble factors and cell-associated receptors that can recognize pathogens and lead to effector functions, including opsonization, phagocytosis, coagulation cascades and expression of antimicrobial peptides [2], [3].

Pathogen recognition functions that involve protein-carbohydrate interactions are mostly mediated by both soluble and cell-associated lectins, which are critical components of the innate immune responses in both vertebrates and invertebrates [4]. Based on their carbohydrate specificity, divalent cation requirements, presence of conserved sequence motifs in their carbohydrate binding domains (CRDs), and most recently their structural folds, animal lectins have classified into several families such as C-, F-, I- and P- type lectins, ficolins and galectins. Among these, the galectins, formerly known as S-type lectins, constitute a multifunctional albeit structurally conserved family of β-galactoside-binding proteins, which contain one or more CRDs per subunit sharing a conserved sequence motif, and are widely distributed among eukaryotic taxa [5]. In mammals, up to 15 distinct galectins (galectin-1 to 15) have been described that fall into three structural types: proto type, with one CRD per subunit and mostly form non-covalently-bound dimers, the chimera type, also with one CRD and an N-terminal polypeptide extension that enables subunit oligomerization into trimers and pentamers, and the tandem repeat type, in which two CRDs are covalently joined by a linker peptide [6].

Initially described as mediating embryogenesis and development by binding to endogenous glycans, in recent years galectins have been shown as key regulators of immune homeostasis [7]–[12]. More recently, however, the elucidation of their roles in multiple aspects of innate immune responses of vertebrates has included the direct recognition of microbial pathogens and function as pattern recognition receptors [13]. Thus, in addition to recognizing endogenous ‘self’ glycans, galectins can recognize microbe-associated molecular patterns consisting of similar ‘non-self’ carbohydrate moieties [14]. In invertebrates, galectins are thought to participate in biological processes that are common to those from vertebrates based on ‘self’ and ‘non-self’ recognition described above. However, relatively few studies about the biological roles of galectins in invertebrates have been reported. A galectin (*Cv*Gal1) of unique CRD organization from the eastern oyster *Crassostrea virginica*, can recognize a large number of environmental bacterial species and strains, as well as phytoplankton microalgae [15]. Furthermore, *Cv*Gal1 is up-regulated by infectious challenge with trophozoites from the parasite *Perkinsus marinus*, and promotes their phagocytosis by binding both to the hemocyte surface (‘self’) glycans as well as the parasite surface (‘non-self’) sugars [15], [16]. The parasite glycans recognized by *Cv*Gal1 are structurally different from those recognized on the oyster hemocytes [16], [17], and as an example of carbohydrate-based molecular mimicry the interactions with *Cv*Gal1 facilitate host entry by the parasite [13], [14], [18]. A similar galectin (*Mc*Gal) that is upregulated by microbial challenge and can recognize microbial pathogens and parasites was later described in the Manila clam *Ruditapes philippinarum* [19].

As the consumer demand for farmed shrimp has continued to increase worldwide, so has the rate of infectious disease in the aquaculture industry settings, and there is an urgent need to address mechanistic questions concerning the shrimp immune responses to microbial pathogens. Although various aspects of the roles of antimicrobial peptides, C-type lectins, and other immune factors have been characterized in considerable detail [20]–[22], galectins have been described in only a few crustacean species [23], and no functional studies have been reported so far. In this study, we identified and characterized a galectin from the kuruma shrimp *Marsupenaeus japonicus,* which we designated *Mj*Gal (*Marsupenaeus japonicus* galectin). The expression of *Mj*Gal is up-regulated upon bacterial challenge, and the protein bind to bacteria through the recognition of lipoteichoic acid (LTA) or lipopolysaccharide (LPS). By also binding to the shrimp hemocyte surface, *Mj*Gal functions as an opsonin for microbial pathogens, promoting their phagocytosis and clearance from circulation, and thereby contributing to the shrimp’s immune defense against infectious challenge.

## Materials and Methods

### Reagents, Virus and Bacterial Strains

Peptidoglycan (PGN) (*Micrococcus luteus*), lipoteichoic acids (LTA) (*Staphylococcus aureus*) and lipopolysaccharide (LPS) (*Escherichia coli*) were from Sigma (St. Louis, MO, USA). All other reagents and chemicals were molecular biology grade or obtained at the highest purity available from Sangon Biotech (Shanghai, China). *Staphylococcus aureus*, *E. coli*, *Bacillus megaterium*, *B. subtilis*, *B. thuringiensis*, *Klebsiella pneumoniae*, *Pseudomonas aeruginosa* and *Vibrio anguillarum* were obtained from Shandong University Organism Culture Collection (SDMCC), and cultured in our laboratory in Luria-Bertani medium.

### Animals

Healthy kuruma shrimp (*Marsupenaeus japonicus*) with average weight of 6–10 g each were obtained from a shrimp market in Jinan, Shandong province, China. The shrimp feeding with the commercial diet daily were acclimated in aerated artificial seawater (Salinity 22%, w/v) at 22°C for two days before use.

### Immune Challenge

For the bacterial challenge, the cultured *V. anguillarum* were collected by centrifugation at 5,000 *g* for 3 min, washed trice by sterile phosphate buffered saline (PBS, 137 mM NaCl, 2.7 mM KCl, 10 mM Na_2_HPO_4_, 2 mM KH_2_PO_4_, pH 7.4), resuspended in PBS, plated for colony counting, and the bacterial suspension adjusted to 2×10^8^ cfu/ml. Fifty µl of the bacterial suspension containing 1×10^7^ cfu were injected into each shrimp intramuscularly at the fourth abdominal segment. Control animals were injected with the same volume of PBS alone.

The experimental and control animals (at least three shrimp at each time point) were bled at 0, 2, 6, 12, 24, 48 h after challenge. Hemolymph was drawn from the ventral sinus using a 5 ml sterile syringe fitted with a 26-gauge needle, and collected into 1 ml of pre-cooled anticoagulant (450 mM NaCl, 10 mM KCl, 10 mM EDTA, 10 mM HEPES, pH 7.45) at a ratio of 1∶1, and immediately centrifuged at 600 *g* for 10 min at 4°C to separate the hemocytes. Subsequently, the animals were euthanized and dissected, and heart, hepatopancreas, gills, stomach and intestine tissues were collected. The tissue samples and hemocyte pellet were immediately processed for RNA extraction.

### cDNA Cloning and Sequence Analysis of the Shrimp Galectin (*Mj*Gal)

A sequence analysis of the shrimp EST database generated in our laboratory enabled the identification of a transcript that showed homology to members of the galectin family, and that we designated as *MjGal*. To clone the *MjGal* full-length transcript, total RNA was extracted from hepatopancreas and gills of shrimp using the Unizol reagent (Biostar, Shanghai, China). The cDNA synthesis was conducted with the SMART cDNA kit (BD Biosciences Clontech) following the manufacturer’s instructions. *MjGal* was amplified with a set of primers designed on the identified EST sequence as follows: Gal F (5′-AAA ACA ACC CAG ACA GCA GTC ATG T-3′) and Gal R (5′-GTA TTT CAA GGC CGT ATA AAC CAC TGC A-3′). The PCR product was sequenced, and BLAST (http://blast.ncbi.nlm.nih.gov/Blast.cgi/) was used for a similarity sequence analysis, ExPASy (http://cn.expasy.org/) to deduce the protein sequence, and SMART (http://smart.embl-heidelberg.de) to predict the domain organization. Finally, the *MjGal* sequence was aligned with representative family members from several invertebrate species and a phylogenetic analysis was carried out using the MEGA 5.05 program [24].

### Semi-quantitative RT-PCR and Quantitative Real Time PCR (qRT-PCR)

The tissue localization of the *MjGal* transcripts (in the unchallenged shrimp) was examined by semi-quantitative RT-PCR with the primers Gal RT F (5′-CCA ACC CTG CTA CTA TGA CC-3′) and Gal RT R (5′-ATC CAC TGC TCC CTG ACC-3′). The PCR amplification program was as follows: 94°C for 5 min followed by 30 cycles at 94°C for 30 s, 58°C for 30 s, 72°C for 20 s, and finally 72°C for 10 min. β-actin was used as internal control with primers β-actin F (5′-AGT AGC CGC CCT GGT TGT AGA C-3′) and β-actin R (5′-TTC TCC ATG TCG TCC CAG T-3′). The *MjGal* expression profiles upon *V. anguillarum* challenge were investigated by quantitative real-time PCR (qRT-PCR) with the same primers used for the semi-quantitative RT-PCR. The qRT-PCR was carried out with SYBR Premix Ex Taq (Takara, Japan) following the manufacturer’s instructions, in a real-time thermal cycler (Bio-Rad, USA). The total volume was 10 µl each including 5 µl of Premix Ex Taq, 2 µl of each primer (1 mM) and 1 µl of cDNA (1/100 diluted). The qRT-PCR was performed at 95°C for 5 min, followed by 40 cycles at 95°C for 10 s, 68°C for 50 s, and finally, melting from 60°C to 95°C. Every sample was tested in triplicate and the *MjGal* expression levels examined by the comparative C_T_ method, and the expression patterns upon infectious challenge analyzed by 2^−ΔΔ*C*T^_._ The unpaired sample *t*-test was used for statistic analysis, and the significant difference was accepted at *P*<0.05.

### Recombinant Expression of *Mj*Gal and Mutant *Mj*Gal^Δ102–106^

For recombinant expression of *Mj*Gal, the sequence was amplified using the primers Gal exF (5′-TAC TCA CAT ATG (*Nde* I) ATG TCA GCA CCA GTG TAC AAC-3′) and Gal exR (5′-TAC TCA CTC GAG (*Xho* I) TCA TAT ACC CTC CTC CAG CAA GGG-3′). The amplicon and the expression vector [pET30a (+)] were digested with *Nde* I and *Xho* I, and ligated at 22°C for at least 1 h. Recombinant plasmids were transformed into *E. coli* BL21 (DE3), cultured overnight, and transferred into the fresh LB medium (1∶100) containing 0.1 mg/ml kanamycin and cultured for 2–3 h at 37°C. As the A_600_ of the culture reached OD 0.6, *Mj*Gal expression was induced by isopropyl β-D-1-thiogalactopyranoside (IPTG) at a final concentration of 0.5 mM, followed by incubation for 4 h at 37°C, after which the bacteria were collected by centrifugation at 6,000 *g* for 15 min, and resuspended in 20 ml of PBS containing 0.2% TritonX-100. The bacteria were lysed by sonication, centrifuged at 12,000 *g* for 15 min, the supernatant removed, and the pellet containing the inclusion bodies washed twice with buffer A (50 mM Tris-HCl, pH 8.0, 5 mM EDTA) followed by buffer B (50 mM Tris-HCl pH 8.0, 2 M urea, 5 mM EDTA), and finally dissolved in 20 ml buffer C (0.1 mM Tris-HCl, pH 8.0 10 mM DTT, 8 M urea). Refolding of the recombinant *Mj*Gal (r*Mj*Gal) protein was carried out by one step of dialysis in buffer (0.1 mM Tris-HCl pH 8.0, 5 mM EDTA, 5 mM cysteine) for 8 h at 4°C (1 L, with three buffer exchanges; 24 h total dialysis time). This was followed by centrifugation at 12,000 *g* for 15 min at 4°C to remove insoluble proteins, and the r*Mj*Gal purified by His Bind resin chromatograpy. A recombinant thioredoxin carrying a His tag [Trx-His; from parent vector pET-32a (+)] was expressed and purified following the same protocols, and used as the control protein.

In order to rigorously validate the function of *Mj*Gal, a mutant *Mj*Gal was constructed by deletion of five residues from sites 102 to 106 and recombinant expressed in *E. coli*. Primers GalMtF (5′-GTG ACC ATC CTC TTC AAG ATT GCC GTG AAT-3′) and Gal exR were used for amplifying fragment I, and Gal exFand GalMtR (5′-GGC AAT CTT GAA GAG GAT GGT CAC TTC AAA-3′) were used for amplifying fragment II. After the two fragments were amplified, the full length of *Mj*Gal^Δ102–106^ DNA fragment was amplified by the overlapping PCR using the two purified DNA fragments as the templates. And it was ligated to *Nde* I and *Xho* I site of expression vector [pET30a (+)]. Expression and purification of the recombinant mutant *Mj*Gal were performed following the same way as the wild type *Mj*Gal.

### Western Blot Analysis

Samples were run on 12.5% SDS-PAGE [25], the proteins transferred onto a nitrocellulose membrane, blocked with 3% nonfat dry milk in 10 ml TBS (10 mM Tris-HCl, 150 mM NaCl, pH 8.0) for 1 h at RT with gentle shaking, and incubated with the mouse anti-histidine (anti-His) antibody (ZSGB Bio, Beijing, China, 1∶3000 dilution in block reagent) for at least 4 h at RT. The membrane was washed with TBST (0.1% Tween-20 in TBS) 3 times for 10 min each, and incubated with alkaline phosphatase-conjugated horse anti-mouse IgG (ZSGB Bio, Beijing, China, 1∶5000 dilution in block reagent) for at least 3 h. This was followed by washing for 3×10 min with TBST and 1×10 min with TBS, and signal was visualized by solution [45 µl Nitroblue Tetrazolium (75 mg/ml), and 35 µl 5-Bromo-4-chloro-3-Indolyl Phosphate (50 mg/ml) in 10 ml TBS] in the dark.

### Binding Activity of r*Mj*Gal to Bacteria

Gram-positive (G^+^) bacteria (*B. megaterium, B. subtilis*, *B. thuringiensis* and *S. aureus*) and Gram-negative (G^−^) bacteria (*E. coli, K. pneumoniae, P. aeruginosa* and *V. anguillarum*) were tested for binding by *Mj*Gal following the method previously described [26]. Bacteria cultured in LB medium (1% Tryptone, 1% NaCl and 0.5% yeast extract) were harvested in the mid-logarithmic phase by centrifugation at 6,000 *g* for 10 min, washed twice with TBS, and re-suspended with TBS to OD_600_ = 1.0. Purified r*Mj*Gal (500 µg/ml, 6 µl) was pre-incubated with either lactose (200 mM, 100 µl), glucose (200 mM, 100 µl), LPS or LTA (1 µg/µl, 50 µl), or TBS alone for 1 h, and suspensions of four G^+^ and four G^−^ were added and incubated at room temperature for 1 h. The bacteria were centrifuged, washed four times with TBS, and treated with 7% SDS for 1 min. The eluate was collected by centrifugation at 10,000 *g*, subjected to SDS-PAGE, and the eluted r*Mj*Gal detected by Western blot using an anti-His antibody.

In an alternative experimental format the purified recombinant *Mj*Gal (500 µg/ml; 6 µl) was added to the bacterial suspension (500 µl; 2×10^7^ cells/ml) and incubated for 30 min at RT. The bacteria were recovered by centrifugation, washed four times with TBS as described above, and either lactose (200 mM) or glucose (200 mM; negative control) were added and incubated for 2 min to elute the bound proteins. The eluate was separated by centrifugation at 10,000 *g*. Subsequently, a second elution was carried out with 7% SDS for 1 min, and the eluate separated by centrifugation as described above. The lactose, glucose and SDS eluates were subjected to SDS-PAGE, and the eluted *Mj*Gal detected by Western blot as described above, using an anti-His antibody. The binding activities of r*Mj*Gal^Δ102–106^ and Trx-His to bacteria were examined as controls, using the same protocol.

### Binding of *Mj*Gal and *Mj*Gal^Δ102–106^ to Bacterial Glycans

The binding of *Mj*Gal and *Mj*Gal^Δ102–106^ to bacterial glycans was examined by enzyme-linked immunosorbent assay (ELISA) following a protocol reported earlier [26]. Briefly, each well of the microtiter plate (Corning, USA) was coated with 2 µg of LPS, LTA, or PGN overnight, and blocked with 200 µl of BSA (1 mg/ml in TBS) for 2 h at 37°C. Fifty microliters of purified recombinant *Mj*Gal or *Mj*Gal^Δ102–106^ (20 µg/ml in TBS containing 0.1 mg/ml BSA) were added to each well and incubated at 37°C for 3 h. Controls were the substitution of *Mj*Gal by Trx-His. After washing four times with TBS, 100 µl of mouse anti-histidine (anti-His) antibody (1∶3000 diluted by TBS containing 0.1 mg/ml BSA) were added to each well and incubated at 37°C for 1 h. The wells were washed four times as described above, and the phosphatase-conjugated horse anti-mouse IgG (100 µl of 1/1000 dilution) was added and incubated for 1 h at 37°C. After the washing four times with TBS, the plates were developed by 100 µl p-nitro-phenyl phosphate (1 mg/ml in 10 mM diethanolamine and 0.5 mM MgCl_2_). Absorbance at 405 nm for each well was analyzed by a plate reader (Bio-Tek instruments). The assay was performed in triplicate.

### In vivo Clearance of *Mj*Gal-coated *V. anguillarum*

The in vivo bacterial clearance experiments were performed following methodology reported elsewhere [21]. Briefly, *V. anguillarum* was washed twice with the sterile PBS, and resuspended to the final concentration of 8×10^8^ cfu/ml. The bacterial suspension (500 µl) was mixed with an equal volume of purified r*Mj*Gal (600 µg/ml, 500 µl) and incubated at 28°C for 30 min with gentle mixing. Controls consisted in the substitution of r*Mj*Gal by Trx-His, and PBS alone. The shrimp were divided into three groups with 18 individuals (∼10 g each) in each group. Fifty µl of the r*Mj*Gal-coated bacteria (which included a total of 2×10^7^ cfu of *V. anguillarum* together with 15 µg r*Mj*Gal) or controls (2×10^7^ cfu of *V. anguillarum* together with 15 µg Trx-His, or PBS alone) were injected into the third abdominal segment of each shrimp from experimental or control groups. About 150 µl of hemolymph of each shrimp were collected from the ventral sinus using 5 ml sterile syringe preloaded with 1 ml ice-cold anticoagulant buffer at 5, 15, 30, 60, 120, and 240 min post-injection. The hemolymph samples of three individual shrimp from each group were immediately pooled, serially diluted in ice-cold PBS, and 30 µl of each dilution plated onto 2216E agar plates (0.1% yeast extract, 0.5% tryptone, 0.1% FeCl_3_, 1.5% agar, and 2.4% artificial seawater salt). The plates were incubated overnight at 37°C, and the number of bacterial colonies counted for each of the three experimental and control groups.

### Binding of *Mj*Gal to Shrimp Hemocytes in vitro

Binding of *Mj*Gal to shrimp hemocytes was examined *in vitro* by adapting a cell adhesion assay described elsewhere [27]. The polylysine pretreated glass slides were coated with purified recombinant r*Mj*Gal protein (10 µg/ml, 200 µl) overnight at 4°C, and subsequently blocked with BSA (1 mg/ml, in PBS buffer). Slides coated with Trx-His were used as controls. Hemolymph from the shrimp was collected from the ventral sinus using 5 ml sterile syringe preloaded with ice-cold anticoagulant buffer and then centrifuged at 600 *g* for 10 min at 4°C to collect the hemocytes. After adjusted to 2×10^4^ cell/ml in PBS, 200 µl of the hemocytes suspension was added into the slide and incubated at room temperature for 1 h. Then the unbound hemocytes were washed away with PBS by 3×10 min and then fixed in 1% paraformaldehyde. Finally, the number of attached cells was counted at 400× magnification under microscope (Olympus BX51, Tokyo, Japan). In the control groups, lactose (200 mM) was added onto the r*Mj*Gal-coated slide at room temperature for 1 h before the hemocytes were added. Cell adhesion activity was calculated in comparison with the numbers of fixed hemocytes between r*Mj*Gal-coated slides and the control group. The assays were performed in triplicate.

### Binding of *Mj*Gal to Shrimp Hemocytes in vivo

The binding of *Mj*Gal to shrimp hemocytes was investigated in vivo by injecting r*Mj*Gal into shrimp (50 µg/each) as described above, replacing the r*Mj*Gal with Trx-His, and injecting PBS alone as the controls. Hemolymph was collected from the ventral sinus with a 2 ml sterile syringe preloaded with 1 ml anticoagulant at 30 min post-injection, and the hemocytes were isolated by centrifugation at 600 *g* for 10 min at 4°C. After washing twice with PBS, the hemocytes were fixed with 1% paraformaldehyde in PBS for at least 10 min. The fixed hemocytes (50 µl) were placed onto a glass slide pretreated with polylysine and allowed to settle for 30 min at RT. After blocking with 3% BSA in PBS for 30 min, an anti-His mouse antibody (1/1000 in PBS containing 3% BSA) was added and incubated for 8 h at 4°C, and the slides washed six times with PBS. After blocking with 3% BSA in PBS for 5 min, FITC-conjugated horse anti-mouse IgG (1∶1000 in PBS containing 3% BSA) was added to the glass slide and incubated for 1 h at 37°C in the dark, washed six times with PBS for 10 min each, stained with DAPI (1/1000 dilution in PBS) for 10 min, washed again six times with PBS for 10 min each, and examined under fluorescence microscope (Olympus BX51, Tokyo, Japan).

### Bacterial Opsonization and Phagocytosis in vivo Assay

This assay was performed following a previous report [28]. Briefly, *V. anguillarum* (around 10^9^ cfu/ml) were heat-killed (75°C for 30 min), washed with 0.1 M NaHCO_3_ (pH 9.0), and stained with fluorescein isothiocyanate (FITC) (Sigma) (1 mg/ml in 0.1 M NaHCO_3_) at 25°C for 1 h, with gentle shaking. The labeled bacteria were washed five times with PBS to remove any remaining FITC, resuspended in PBS, and mixed with 500 µg r*Mj*Gal and incubated at for 28°C for 1 h, with gentle shaking. Finally, the bacteria were washed three times with PBS and adjusted to 10^9^ cells/ml. Trx-His replaced the r*Mj*Gal in the controls. Fifty µl of the labeled bacteria coated with the protein were injected into each shrimp, and hemolymph was drawn at 30 min post-injection, and as described above the hemocytes collected by centrifugation and washed twice with PBS, resuspended in paraformaldehyde (1% in PBS), incubated for 30 min at RT, and washed three times with PBS. The fixed hemocytes (50 µl) were placed onto a glass slide pretreated with polylysine and allowed to settle for 30 min at RT. Subsequently, trypan blue (2 mg/ml in PBS) (Amresco) were added to quench the fluorescence of the extracellular (non-phagocytosed) bacteria. After incubation for 30 minutes, the slide was gently washed five times with PBS, and observed under the fluorescence microscope (Olympus BX51, Tokyo, Japan). Hemocytes (at least 100/slide) were counted at 200× magnification. The phagocytic percentage (PP) was calculated as follows: PP =  (number of cells ingesting bacteria/number of cells observed)×100%. The assay was conducted in triplicate. The data statistically analyzed using *t*-test, and significant differences between *Mj*Gal and control groups were accepted at *P*<0.05.

### Silencing of *Mj*Gal Expression by RNA Interference (RNAi) and Bacterial Clearance Assay

#### Preparation of DsRNA

Selected stretches (546 bp) of *MjGal* DNA were amplified using the primers: RNAi F (5′-TAA TAC GAC TCA CTA TAG G TTT CTC CTG GAC GGA TTG CTC AT-3′) and RNAi R (5′-TAA TAC GAC TCA CTA TAG G CTG CTT GCT GCT GGT AGG GCT GAT T-3′). For use as controls, the primers: GFPi F (5′-TAA TAC GAC TCA CTA TAG G TGG TCC CAA TTC TCG TGG AAC-3′) and GFPi R (5′-TAA TAC GAC TCA CTA TAG G CTT GAA GTT GAC CTT GAT GCC-3′) were used to amplify GFP DNA. The sequences underlined correspond to the T7 promoter. The amplification products were extracted and precipitated with phenol/chloroform and ethanol, and used to synthesize dsRNA. The transcription was performed as follows: 6 µg DNA template, 2.4 µl 10 mM A/U/C/GTP each, 80 U RioboLock RNase Inhibiter, 20 µl 5× transcription buffer and 80 U T7 RNA polymerase were mixed (Fermentas, USA). Then RNase-free water was added to a total volume of 50 µl, and incubated overnight at 37°C. To remove the template, 8 U RNase-free DNase I, 10 µl 10× Reaction Buffer (Fermentas, USA), 32 µl RNase-free water were added and incubated at 37°C for 30 min. After extraction and precipitation with phenol/chloroform and ethanol respectively, dsRNAs were dissolved in 50 µl RNase-free water. The amount and purity of dsRNAs were analyzed by 2% agarose gel electrophoresis and a K5500 micro-spectrophotometer (Beijing Kaiao, China) was used to quantify the amount of dsRNAs.

#### RNAi assay

Shrimp (∼10 g) were divided into two groups, and injected with either 40 µg *Mj*Gal dsRNAs or GFP dsRNAs (controls). A similar second injection of dsRNA was performed 24 h later. Before conducting the clearance assay, three shrimp of each group were selected and the RNA of the hemocytes and gills were isolated to synthesize cDNA and confirm silencing of *MjGal* expression. A pair of primers: Check F (5′-TTT CTC CTG GAC GGA TTG CT-3′) and Check R (5′-CCT GCT GCC ATC CCC AGT AAT C-3′) were used for RT-PCR amplification.

#### Bacterial clearance assay

*V. anguillarum* (2×10^7^ cells) were injected into shrimp 12 h after the dsRNA second injection. At 5, 15, 60 min of post-infection, hemolymph was collected from the two groups, and serially diluted immediately. Fifty µl of each dilution were plated onto 2216E agar plates, incubated at 37°C overnight, and the colonies counted as described above.

## Results

### CDNA Cloning and Phylogenetic Analysis of *Mj*Gal

The full length of *MjGal* was obtained by random sequencing (GenBank accession No. JQ804931). It contained an open reading frame (ORF) of 1002 bp encoding a protein of 333 amino acids, and contained a galactoside-binding lectin domain. The theoretical *p*I and molecular mass of mature *Mj*Gal was 7.7 and 33.5 kDa, respectively.

**Figure 1:**
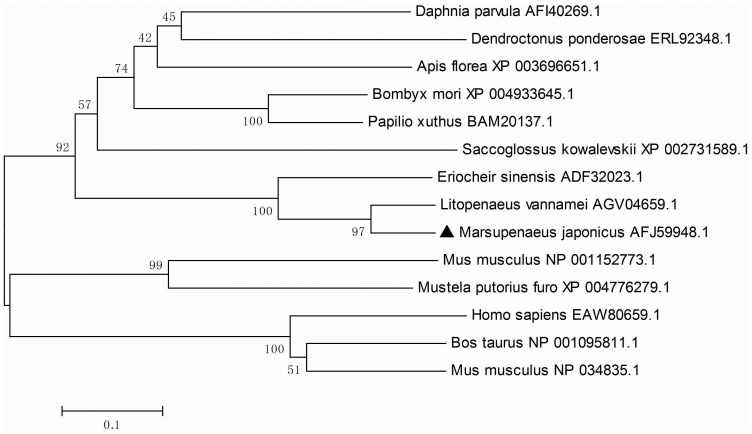
Phylogenetic analysis of *Mj*Gal. The tree was constructed with the MEGA 5.05 software. One thousand bootstraps were used to check the repeatability of the results. The galectin sequences of selected invertebrate and vertebrate species were obtained from NCBI GenBank. *Mj*Gal is labeled with a solid triangle.

BLAST analysis showed that *Mj*Gal shares high identity with galectins from crustaceans such as the Pacific white shrimp, *Litopenaeus vannamei* (AGV04659, 89%) and the Chinese mitten crab *Eriocheir sinensis* (ADF32023, 58%), but to a lesser degree to the galectin from the water flea *Daphnia pulex* (EFX86664.1, 28%). *Mj*Gal also showed similarity to galectins from insects, such as those from the leaf-cutter ant *Acromyrmex echinatior* (EGI70288.1, 34%), the honeybee *Apis florea* (XP_003696651.1, 36%), the jumping ant *Harpegnathos saltator* (EFN89847.1, 34%), and the bumble bee *Bombus impatiens* (XP_003493369.1, 33%). The *Mj*Gal sequence displayed similarity with some mollusk galectins, such as those from the European oyster *Ostrea edulis* (ADF80416.1, 34%), the clam *Ruditapes philippinarum* (ACA09732.1, 34%) and the Pacific oyster *Crassostrea gigas* (EKC40502.1, 28%). A phylogenetic tree (MEGA 5.05 software) based on the amino acid sequences of selected galectins (**Fig. 1**) revealed that *Mj*Gal clustered with galectins from arthropods and the hemichordate *Saccoglossus kowalevskii,* whereas all galectins from vertebrates formed a separate cluster.

### *Mj*Gal was Up-regulated by *V. anguillarum* Challenge

**Figure 2:**
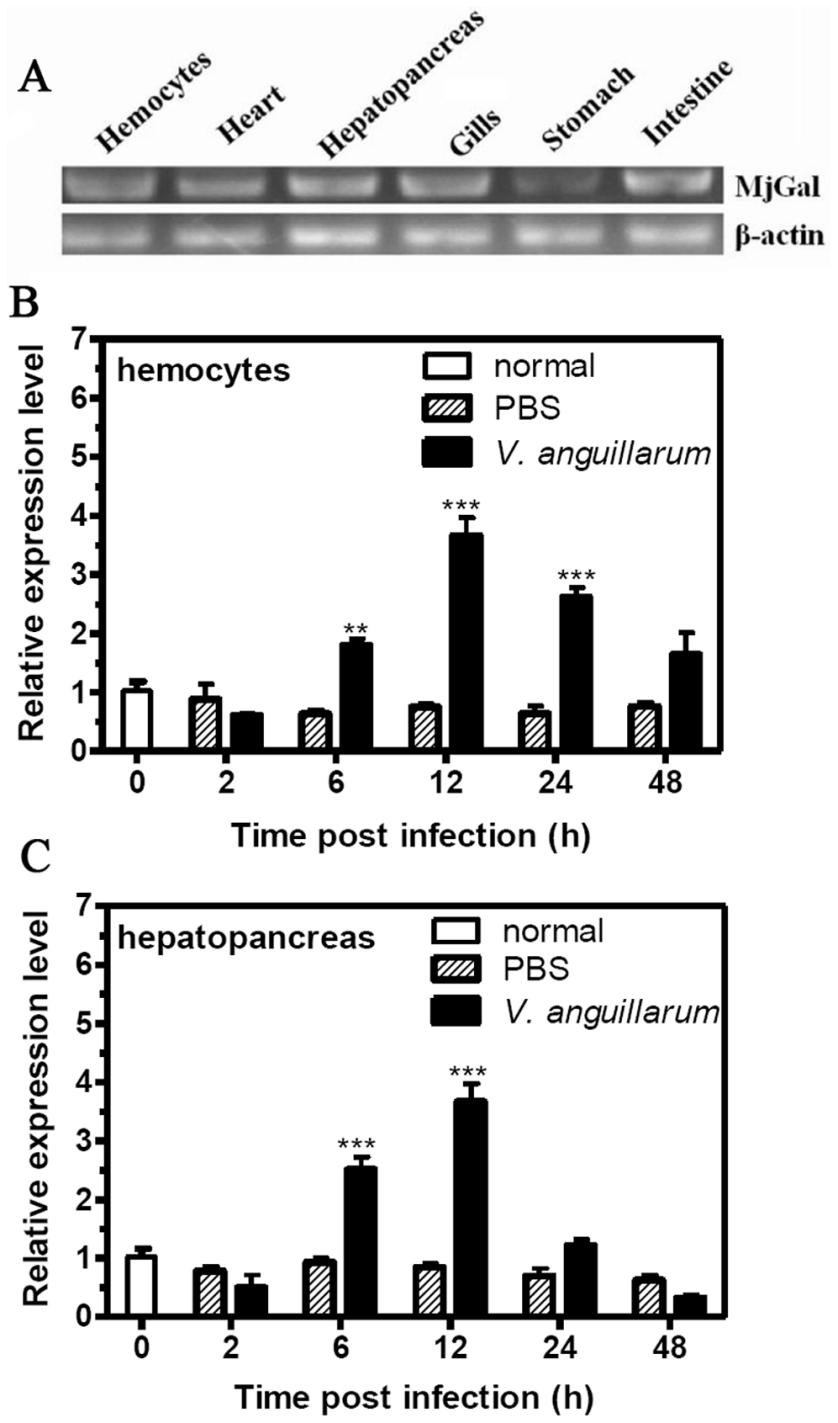
Temporal and spatial expression of *MjGal*. Total RNAs from different tissues of unchallenged and *V. anguillarum* or PBS challenged shrimp were reverse transcribed to cDNAs that served as templates for PCR amplification. (A) Expression of *MjGal* in different tissues of naïve animals as assessed by RT-PCR. The time-course expression of *MjGal* was upon *V. anguillarum* challenge was analyzed by qRT-PCR in hemocytes (B) and hepatopancreas (C), with β-actin serving as the reference gene. The histograms show the statistical analysis of the quantitative real-time PCR results. The asterisks indicate significant differences (**P<0.01, ***P<0.001) between the *V. anguillarum*-challenged and PBS-injected group. Error bars represent ± SD of three independent PCR amplifications and quantifications.

Expression of *MjGal* mRNA in various tissues was examined by RT-PCR. *MjGal* was detected in all the tested tissues including hemocytes, heart, hepatopancreas, gills and intestine with a high level, and at a relatively lower level, in the stomach (**Fig. 2A**). A time-course assessment of *MjGal* expression upon *V. anguillarum* challenge showed that *MjGal* expression was up-regulated about 4-fold both in the hemocytes (**Fig. 2B**) and hepatopancreas (**Fig. 2C**) over the PBS-control at 12 h post-injection, and these were recovered at 48 h post-injection (**Fig. 2B and C**).

### Recombinant *Mj*Gal Bound to but did not Agglutinate Bacteria

**Figure 3:**
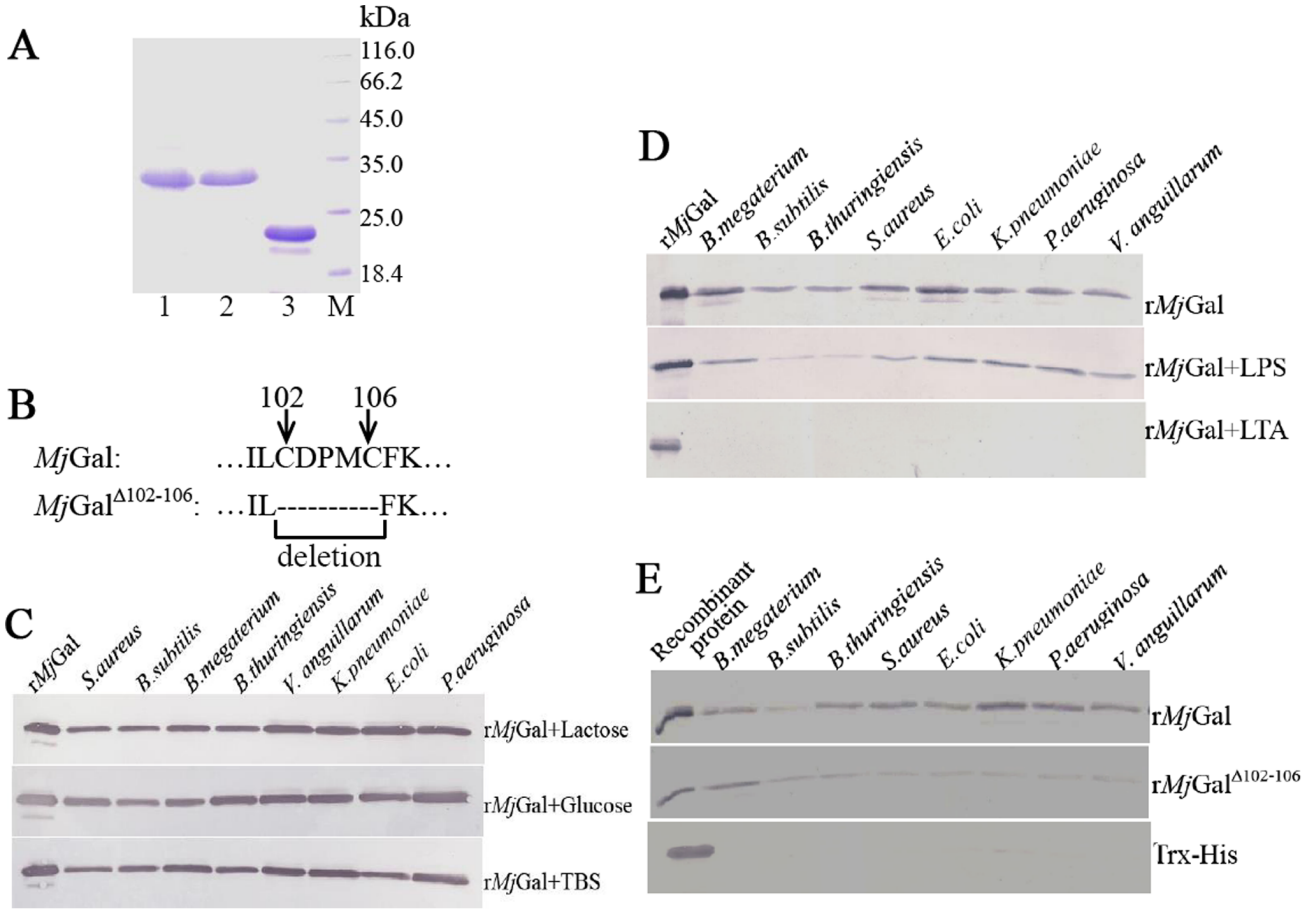
Expression of recombinant *Mj*Gal (r*Mj*Gal), r*Mj*Gal^Δ102–106^ and their bacteria binding assay by Western blot. (A) Lane 1, purified r*Mj*Gal; lane 2, purified r*Mj*Gal^Δ102–106^; lane 3, purified Trx-His which was used as the control;lane 4, protein markers. (B) Schematic diagram of the five deleted residues (Cys102-Cys106) in mutant *Mj*Gal^Δ102–106^. (C) Purified r*Mj*Gal (500 µg/ml) was pre-incubated with either lactose (200 mM), glucose (200 mM), or TBS alone for 1 h, and suspensions of four Gram-positive (G^+^) bacteria (*S. aureus*, *B. subtilis*, *B. megaterium* and *B. thuringiensis*) and four Gram-negative (G^−^) bacteria (*V. anguillarum*, *K. pneumoniae*, *E. coli* and *P. aeruginosa*) were added and incubated at room temperature for 1 h. The bacteria were centrifuged, washed four times with TBS, and treated with 7% SDS for 1 min. The eluate was collected by centrifugation, subjected to SDS-PAGE, and the eluted r*Mj*Gal detected by Western blot using anti-His antibody. (D) The purified r*Mj*Gal was pre-incubated with excess LPS or LTA before the bacteria were added, and the binding activity for bacteria was tested by western blot. (E) Purified r*Mj*Gal^Δ102–106^ or Trx-His were also used as controls for the bacteria binding activity.

*Mj*Gal and *Mj*Gal^Δ102–106^ were recombinant expressed in *E. coli* BL21 (DE3) and purified by His Bind resin chromatography. The molecular mass of the recombinant *Mj*Gal and *Mj*Gal^Δ102–106^ were consistent to the expected molecular mass (**Fig. 3A and B**).

Recombinant *Mj*Gal bound to the various microorganisms tested, including four G^+^ bacteria (*S. aureus, B. thuringiensis, B. subtilis* and *B. megaterium*) and four G^−^ bacteria (*E. coli, V. anguillarum, K. pneumoniae* and *P. aeruginosa*). The results showed that *Mj*Gal strongly bound to different bacteria, especially the G^−^ bacteria. To test whether pre-exposure of r*Mj*Gal to a galectin carbohydrate ligand could prevent the binding of r*Mj*Gal to the bacteria, the purified r*Mj*Gal (500 µg/ml) was pre-incubated with lactose (a typical galectin ligand) or glucose (as the negative control), and the bacteria were added, incubated for 1 h, washed, and the bound proteins eluted with 7% SDS. As shown in **Fig. 3C**, the pre-incubation with either lactose or glucose had no effect on the binding of r*Mj*Gal to the selected bacteria. An alternative experimental format was tested to examine whether lactose could displace the r*Mj*Gal bound to bacteria. For this, the G^+^ and G^−^ bacteria were incubated with the purified r*Mj*Gal (500 µg/ml) for 1 h, washed and eluted with either 200 mM lactose or glucose. The sugar failed to displace any bound r*Mj*Gal, but the protein could be readily eluted from the bacteria with 7% SDS (Data not shown). Pre-incubation of r*Mj*Gal with LPS and LTA showed that LPS partially inhibited the binding activity of r*Mj*Gal to bacteria, whereas LTA completely inhibited the binding activity (**Fig. 3D**). The binding activity of r*Mj*Gal^Δ102–106^ for bacteria was much weaker than that of the wild type r*Mj*Gal (**Fig. 3E**). Trx-His did not show binding activity for bacteria (**Fig. 3E**). Taken together, the results showed that r*Mj*Gal bound strongly to the selected bacterial species tested, that the binding takes place via the CRD, and that although lactose could not inhibit or displace the binding of the galectin to bacteria, both LTA and LPS could function as effective binding inhibitors. It is noteworthy that an agglutination assay with r*Mj*Gal and the bacterial species described above showed no agglutination for any of the bacteria tested (Data not shown).

### r*Mj*Gal Bound to LPS and LTA Strongly and r*Mj*Gal^Δ102–106^ Bound to LPS and LTA Weakly

**Figure 4:**
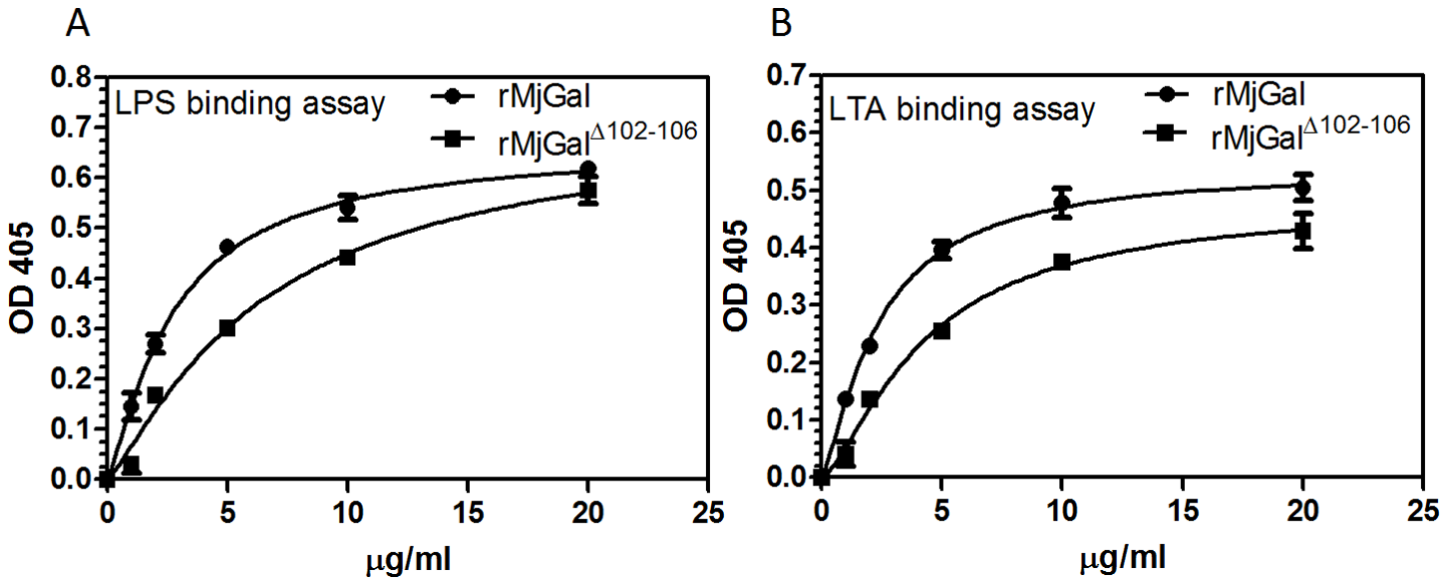
r*Mj*Gal strongly bound to lipopolysaccharide (LPS), lipoteichoic acid (LTA). r*Mj*Gal^Δ102–106^ weakly bound to LPS, LTA. Three polysaccharides LPS, LTA and peptidoglycan (PGN) were used in this binding assay. Microtiter plates were coated with 2 µg of LPS (A) or LTA (B), and incubated with increasing concentrations of r*Mj*Gal or r*Mj*Gal^Δ102–106^ (0, 1, 2, 5, 10, 20 µg/ml). Binding of r*Mj*Gal or r*Mj*Gal^Δ102–106^ was detected using anti- Histidine antibody. Data shown are the mean±SEM derived from three repeats. No binding to PGN could be detected at any of the r*Mj*Gal concentrations tested (results not shown).

The enzyme-linked immunosorbent assay (ELISA) was performed to detect whether r*Mj*Gal bound to polysaccharides (LPS, PGN and LTA). The results showed that r*Mj*Gal could bind to polysaccharides, including LPS and LTA, but not PGN (data not shown). The binding of *Mj*Gal to polysaccharides was saturable (**Fig. 4A and 4B**). In ELISA, the r*Mj*Gal^Δ102–106^ showed a relatively weaker binding activity to LPS and LTA compared to the wild type r*Mj*Gal.

### r*Mj*Gal Enhanced the Bacterial Clearance Activity in vivo

**Figure 5:**
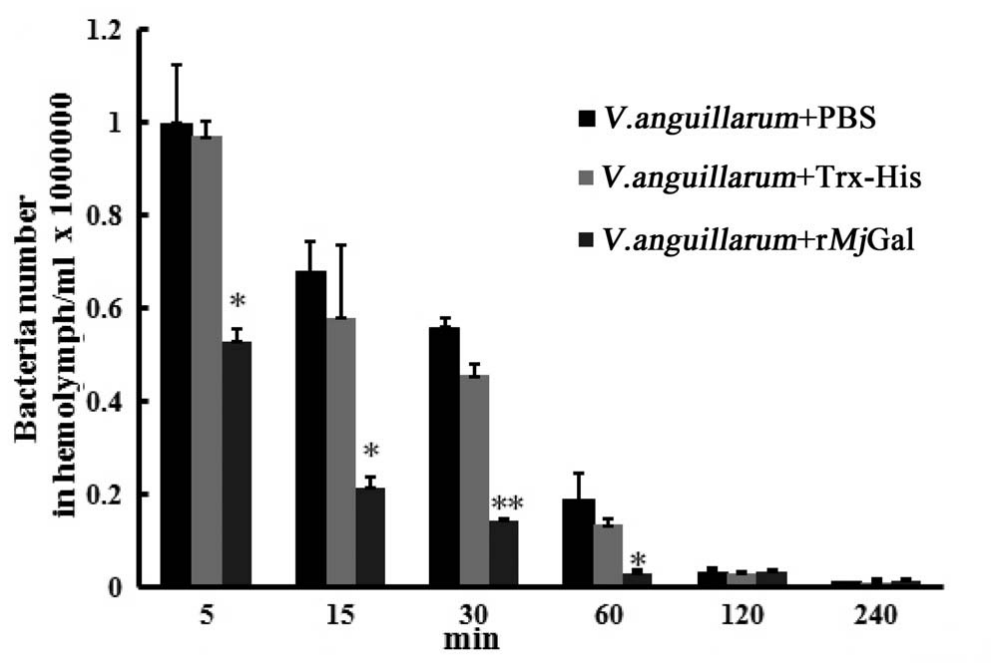
The activity of r*Mj*Gal in the clearance of *V. anguillarum*. Bacteria (2×10^7^ cells) pre-incubated with r*Mj*Gal (15 µg) were injected into the shrimp. Hemolymph was collected, diluted 1∶1000, and plated on 2216E agar plates. Bacterial colonies were counted after 37°C cultivation for 12 h. Results represent as the number of bacteria from three independent repeats and expressed as mean values ±S.D. Asterisks indicate significant differences (*P<0.05, **P<0.01, ***P<0.001).

To investigate the clearance activity of r*Mj*Gal, the shrimp were injected with *V. anguillarum* that had been pre-incubated with either r*Mj*Gal, Trx-His, or PBS, and the bacteria remaining in the hemolymph were counted at different time points after injection. The bacterial counts in the r*Mj*Gal-coated group were significantly lower as compared with two control groups. Further, the two control groups took significantly longer time to reach the similar clearance level of the experimental group (**Fig. 5**). The results suggested that r*Mj*Gal could enhance the clearance activity of *V. anguillarum* in vivo.

### r*Mj*Gal Bound to the Shrimp Hemocyte Surface both in vitro and in vivo

**Figure 6:**
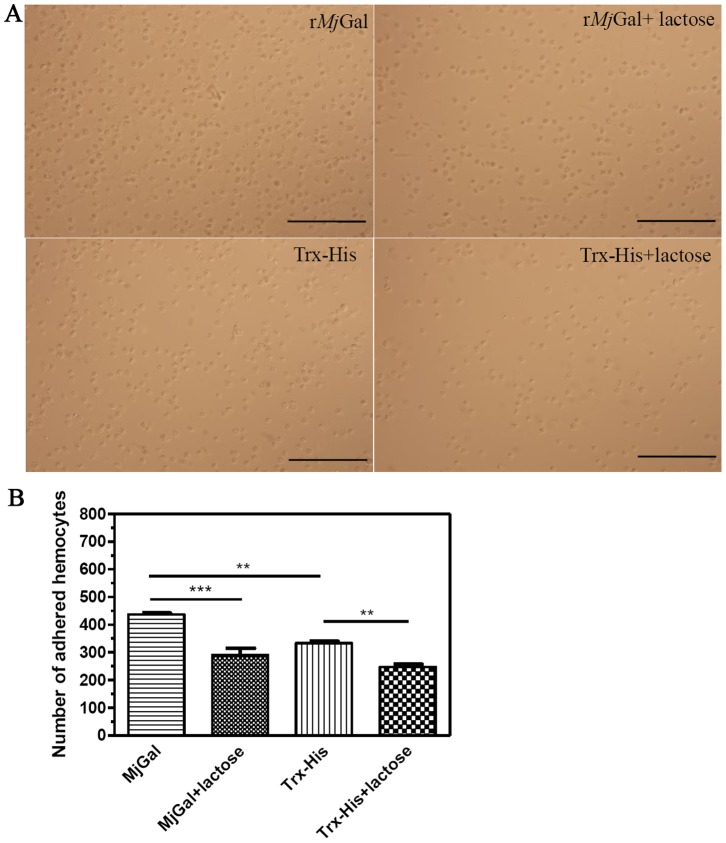
*In vitro* binding of r*Mj*Gal to shrimp hemocytes. (A) Shrimp hemocytes (2×10^4^ cells/ml) were added to the slide glass pre-coated with r*Mj*Gal, r*Mj*Gal with lactose, Trx-His, or Trx-His with lactose. One hour later, the slide was washed three times with PBS and observed under the microscope. The number of hemocytes counted at 400× magnification under microscope. Figures showed the hemocytes which were adhered to the slides after the washing. Assays were performed in triplicate with three different hemocytes suspensions from each shrimp. Bar = 100 µm. (B) Statistical analysis of the data from (A) using GraghPad software. Significant differences were analyzed by one way ANOVA by a Trukey’s multiple comparison test and indicated by asterisks (**P<0.01, ***P<0.001). The bar was 100 µm.

To examine the possible mechanism responsible for the above observation, the binding of r*Mj*Gal to shrimp hemocytes was assessed in vitro by a standard cell adhesion assay in glass slides that had been pre-coated with r*Mj*Gal or control protein. The results revealed that a significantly higher number of hemocytes adhered to the r*Mj*Gal-coated slide than to the Trx-His-coated slide. The addition of lactose reduced significantly the number of hemocytes that adhered to the r*Mj*Gal-coated slide as compared to the control group (**Fig. 6A and 6B**). A reduction in hemocyte adhesion in the Trx-His-coated slide by addition of lactose was possibly due to inhibition of the hemocyte endogenous galectin secreted and bound to the surface of the hemocytes that might partially contribute to hemocyte binding to the slide.

**Figure 7:**
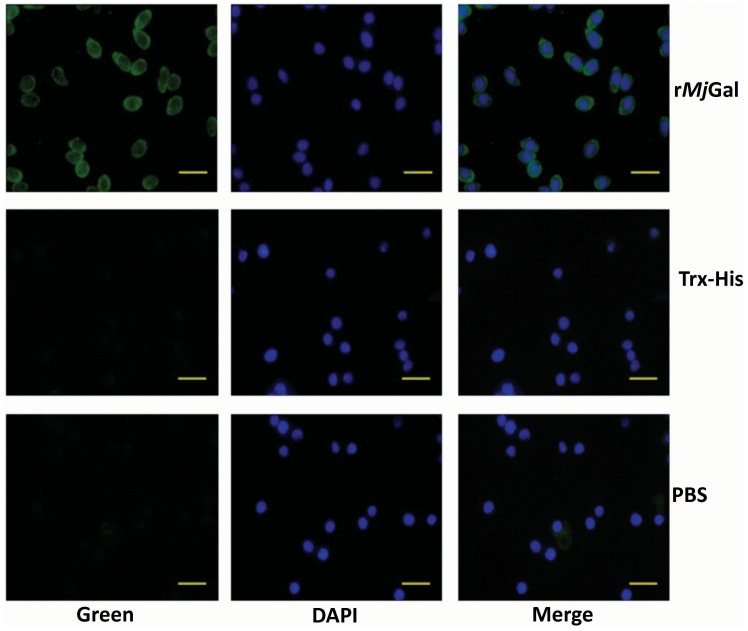
*In vivo* binding of r*Mj*Gal to shrimp hemocytes. r*Mj*Gal, Trx-His, or PBS were injected into shrimp and the hemocytes were collected and analyzed by immunohistochemistry. The left panel indicated the binding signal detected by the anti-Histidine antibody for the recombinant protein (Green), the middle panel showed the hemocyte nucleus location (Blue), and the right panel showed the merge of previous two panels. Bar = 20 µm.

To verify if the *in vitro* r*Mj*Gal-shrimp hemocyte interactions above also occurred *in vivo*, shrimp were injected with r*Mj*Gal, and after 30 min the hemocytes collected as described above, and the presence of surface-bound r*Mj*Gal examined by immunocytochemistry. The images in **Fig. 7** show the green signal on the edge of the hemocytes collected from shrimp injected with r*Mj*Gal, and a significantly weaker signal in the control group. These results indicated that the r*Mj*Gal can bind to the hemocyte surface both *in vitro* and *in vivo*.

### *Mj*Gal Promoted Phagocytosis of Bacteria by Hemocytes

**Figure 8:**
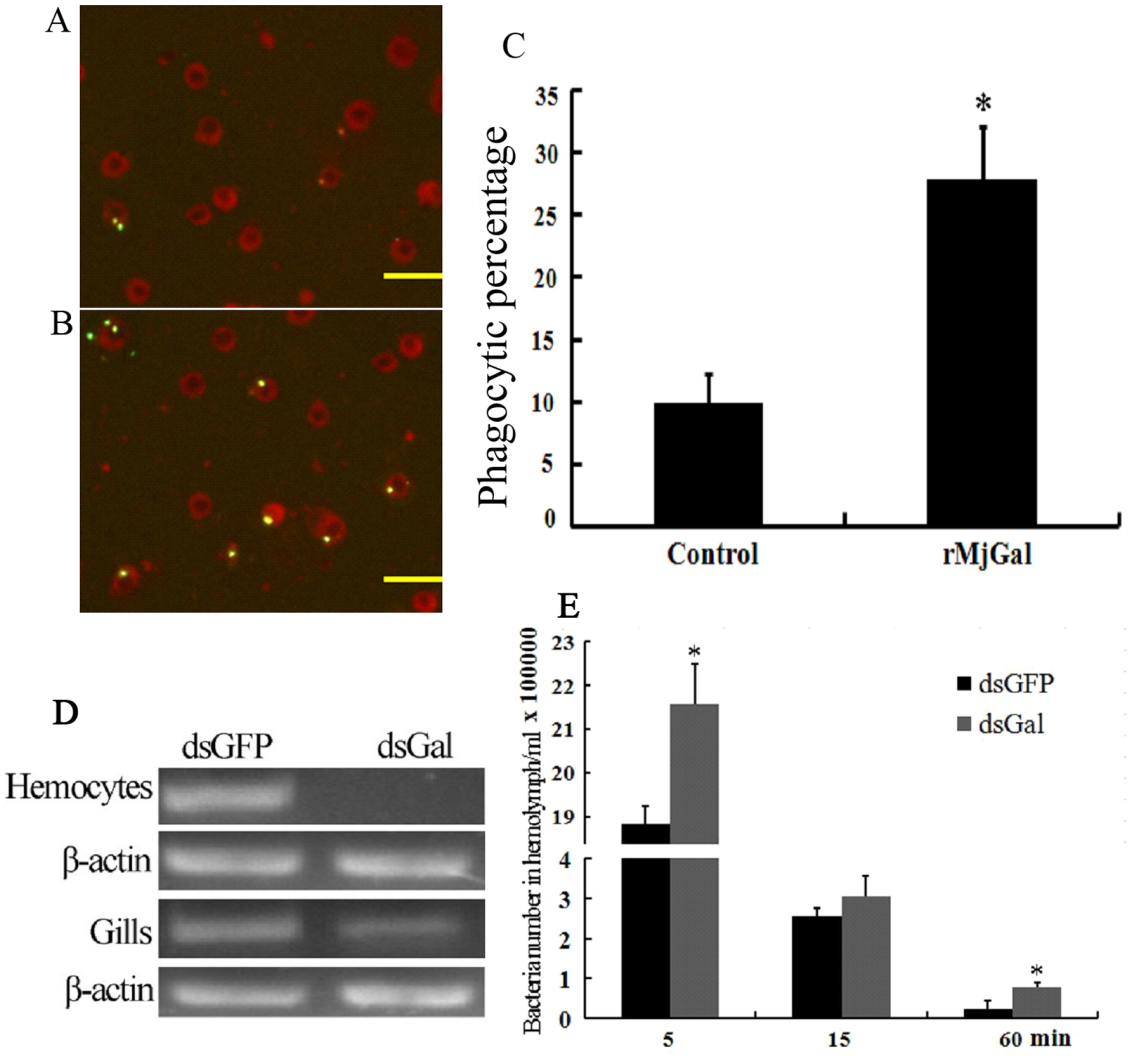
*Mj*Gal promoted bacterial clearance from hemolymph. Bacteria phagocytosis assay: fluorescently labeled *V. anguillarum* (5×10^7^ cells) were coated with either Trx-His (A, the control) or r*Mj*Gal (B) and injected into shrimp. The hemocytes were collected after 30 min and placed onto the glass slides. Subsequently, trypan blue solution (2 mg/ml PBS) (Amresco) was added to quench the fluorescence of non-phagocytosed bacteria. The phagocytosis by hemocytes was observed at 400× magnification, and phagocytosed bacteria counted at 200×magnification under the fluorescence microscope. (A) Hemocytes from shrimp injected with *V. anguillarum* coated with Trx-His. (B) Hemocytes from shrimp injected with *V. anguillarum* coated with r*Mj*Gal. (C) The phagocytic percentages of two groups (*P<0.05). Bar = 50 µm. (D) RNA interference assay: shrimp were injected with dsRNAs twice to silence *Mj*Gal expression, and or with GFP dsRNAs as control. *Vibrio anguillarum* (2×10^7^ cells) were injected into shrimp after the second injection. Hemolymph was collected from the two groups at 5, 15, 60 min post-infection, and serially diluted with PBS. Fifty µl of each sample was plated onto 2216E agar plates and incubated at 37°C overnight. RNA was extracted from hemocytes and gills of two groups, and tested by RT-PCR to confirm silencing of *Mj*Gal expression. (E) The bacterial counts in hemolymph samples collected at 5, 15 and 60 min from shrimp from the ds*Mj*Gal and dsGFP groups.

The above results showed that r*Mj*Gal could bind to the hemocyte surface and to G^+^ and G^−^ bacteria, presumably by binding to LTA and LPS, respectively. In order to further investigate its function upon binding to both the hemocytes and bacteria, an *in vivo* phagocytosis assay was performed. The results revealed that almost 30% of the hemocytes were involved in phagocytosis in the *Mj*Gal coated group, while only 10% in the control group (**Fig. 8C**). As compared with the control group (**Fig. 8A**), hemocytes in test group (**Fig. 8B**) phagocytosed more bacteria. These results suggested that binding of *Mj*Gal to hemocytes and/or bacteria *in vivo* promoted their phagocytosis, and led to propose the hypothesis that *Mj*Gal enhances the clearance of potential microbial pathogens from circulation.

### Silencing of *Mj*Gal Expression Reduced Bacterial Clearance

To test the hypothesis on the role of *Mj*Gal in immune defense based the above observations, RNAi was performed for further functional analysis of *Mj*Gal. Results from RT-PCR demonstrated that that injection of *Mj*Gal dsRNA could significantly inhibit mRNA levels of *Mj*Gal in hemocytes (**Fig. 8D**). The injection of bacteria into the *Mj*Gal-silenced shrimp and control animals that had been injected with GFP dsRNA revealed that the numbers of bacteria in the hemolymph of *Mj*Gal-silenced shrimp were significantly higher than in the GFP dsRNA-injected group at 5 min and 60 min (**Fig. 8E**). These results confirmed that *Mj*Gal plays a key role in the shrimp defense against bacterial infectious challenge.

## Discussion

Although galectins were initially described as developmentally regulated in their expression and playing key roles in early embryogenesis, cell differentiation, and tissue organization, in recent years evidence has accumulated in support of their participation in regulation of immune homeostasis by modulating inflammation and multiple aspects of innate and adaptive immunity, including leukocyte chemotaxis, migration and activation [29], B cell development [9], [30], T cell apoptosis [8], [31], and other regulatory aspects of the immune responses of vertebrates [7]–[12]. Further, during the past few years it has become increasingly clear that galectins also bind glycans on the surface of potentially pathogenic microbes and can function as pattern recognition receptors (PRRs) [13]. Therefore, in vertebrates, galectins can regulate immune functions by binding to both endogenous (‘self’) glycans, and microbe-associated (‘non-self’) glycotopes. Although in invertebrates, galectins are thought to participate in those biological processes that are common to vertebrates, relatively few studies have documented their binding to self and non-self glycans and any role(s) in immune function comparable to those from vertebrates. Moreover, galectins have been described only in a few crustacean species, including the Chinese mitten crab *E. sinensis* (GenBank: ADF32023.1), the Pacific white shrimp, *Litopenaeus vannamei* (GenBank: AGV04659.1), and the water flea *D. pulex* (GenBank: AFI40275.1). These studies, however, have been mostly limited to the identification of galectin transcripts, with no further analysis of their biological role(s). Therefore, the functional characterization of crustacean galectins warrants further investigation. In this study, we identified and functionally characterized for the first time a novel galectin from the kuruma shrimp, *M. japonicus.* The shrimp galectin is upregulated by infectious challenge, and can recognize and opsonize potentially pathogenic bacteria by binding to the hemocyte surface and promoting phagocytosis, and silencing of its expression *in vivo* results in reduced bacterial clearance rates in hemolymph, suggesting that *Mj*Gal plays a key role in immune defense against bacterial infection.

A search of our *M. japonicus* EST database revealed a transcript that we designated *Mj*Gal with homology to members of the galectin family, encoding a 333-amino acid long protein of 33.5 kDa apparent mass that displays a single 127-residue long galactoside-binding lectin domain located in the N-terminal half of the polypeptide chain. The primary structural organization of the transcript indicated that it does not fit the canonical features of the proto or chimera type galectins from vertebrates [14]. Although the MW of *Mj*Gal would suggest that it is related to the latter galectin type, the location of the CRD is opposite to the vertebrate representatives, in which the CRD is located closer to the C-terminal region [14]. In contrast, the organization of the *Mj*Gal transcript is quite similar to the galectin *Dp*Gal-2, from the water flea *D. pulex* that houses a 127-amino acid long CRD linked to a low complexity region. Analysis of the nucleotide and deduced amino acid sequences indicated a closest similarity to galectins identified in the shrimp *L. vannamei* and the crab *E. sinensis*, but in spite of the similar transcript organization, a significantly lower sequence similarity. This may be due to the proposed rapid adaptive evolution of immune genes in *Daphnia* [23] that has resulted galectin sequences that are substantially divergent from other crustacean species. A phylogenetic analysis indicated that *Mj*Gal clustered with galectins from invertebrates and a hemichordate, and was clearly distinct from the vertebrate galectins group.

*MjGal* was expressed in all tissues tested in the naïve animals, and upon infectious challenge, *MjGal* expression was significantly upregulated in both hemocytes and hepatopancreas. In crustaceans, most C-type lectins involved in immune function are expressed in the hepatopancreas and released to the circulation [32]. Furthermore, the crustacean hemocytes are involved in various aspects of surveillance and cellular immune responses [32]. Taken together, these results strongly suggested that *Mj*Gal might be involved in antibacterial responses. This was confirmed by *in vivo* bacterial clearance experiments that showed that bacteria that had been pre-incubated with *Mj*Gal and injected into shrimp were cleared more rapidly from circulation than those exposed to the control protein or buffer alone. Although the role of invertebrate lectins (particularly of the C-type) in pathogen clearance has been demonstrated for several species, particularly insects [32] and mollusks [15], this is the first demonstration in a crustacean species that a galectin can promote bacterial clearance and contribute to immune defense against microbial pathogens.

To elucidate the mechanisms involved in bacterial clearance, we investigated whether *Mj*Gal directly recognized and bound to the bacterial surface. *Mj*Gal bound strongly to both G^+^ and G^−^ bacteria, in a manner that could be partially or totally inhibited by LPS or LTA, respectively. However, although the binding could not be inhibited or outcompeted by lactose, a typical galectin ligand, but could be displaced by a detergent such as SDS. In contrast, *Mj*Gal also bound strongly to the hemocyte surface, but the binding could be inhibited (at least partially) by lactose, thereby confirming that like most galectins described to date, *Mj*Gal binds to galactosyl moieties. Thus, the lack of inhibitory effect of lactose on the binding of *Mj*Gal to bacteria requires alternative explanations. First, due to the bacterial oligosaccharide structures or the architecture of their presentation on the microbial surface, *Mj*Gal may bind with higher affinity to microbial surface glycans relative to lactose, which is not able to outcompete the interactions even at high concentrations. These glycans would be different from those displayed on the hemocyte surface, to which *Mj*Gal would bind in a lactose-inhibitable manner. Second, it is also possible that upon binding of *Mj*Gal to the microbial cell surface, a considerable disruption of bacterial membrane morphology takes place thereby exposing areas that establish secondary interactions (ie hydrophobic) with *Mj*Gal that are different from the initial protein-carbohydrate interactions. Displacement of these additional *Mj*Gal-bacteria interactions would require a detergent such as SDS, as observed in our experiments. The disruption of membrane morphology in both prokaryotic and eukaryotic cells upon binding of galectins has been reported elsewhere [29], [33], [34]. Both human Gal-4 and Gal-8 directly kill BGB+ *E. coli* through recognition of bacterial surface carbohydrates *via* a mechanism that drastically alters membrane integrity and bacterial motility [33].

Solid phase binding experiments showed that both LTA and LPS, but not PGN, can be potential targets for *Mj*Gal-mediated recognition of the surface of G^+^ and G^−^ bacteria, respectively. The binding of bacterial glycans by invertebrate lectins has been described for insect and mollusks, with LPS and PGN being the more frequently recognized targets [13], [35]. Kinetic analysis of the *Mj*Gal binding to the for LPS and LTA suggest that *Mj*Gal affinities for these glycans are different, although it is likely that its capacity for recognition will depend on their density, presentation, and distribution on the microbial surface. This, as well as the detailed structural aspects of the interactions that will enable to explain the different affinities warrants further investigation.

The mutant r*Mj*Gal^Δ102–106^ in which two conserved cysteine residues and three additional residues in the CRD were deleted, showed a relatively weaker binding activity for bacteria, LPS or LTA, than that of the wild type r*Mj*Gal. This confirmed that the binding of r*Mj*Gal to the ligands tested takes place via the five canonical amino acid residues in the CRD, but also suggests that other amino acid residues in an extended CRD might contribute to the lectin-ligand interaction.

The functional outcome of the binding of *Mj*Gal to both microbial pathogens and the shrimp hemocyte surface was the enhancement of phagocytosis of bacteria by the hemocytes, as experimentally demonstrated in vivo. This opsonic effect of *Mj*Gal for bacteria that may have invaded the shrimp internal milieu became the rationale to propose the hypothesis that *Mj*Gal-mediated opsonization might be the mechanism by which *Mj*Gal enhances the clearance of potential microbial pathogens from circulation as initially observed. The oyster galectin *Cv*Gal1 binds to the surface of a variety of microbes as well as the parasite *Perkinsus marinus* and the pre-incubation of hemocytes with and anti-*Cv*Gal1 antibody significantly reduced phagocytosis, thus confirming that hemocyte surface-bound *Cv*Gal1 participated in the uptake of cross-linked microbes [15]. As *Mj*Gal can bind to both the microbial and the hemocyte surface, it is possible that *Mj*Gal functions in a similar way to *Cv*Gal1, as a receptor on the surface of hemocytes or as a soluble opsonin to promote phagocytosis.

The *Mj*Gal RNAi silencing experiments rigorously tested this hypothesis *in vivo*, and revealed the numbers of bacteria in the hemolymph of *Mj*Gal-knockdown shrimp were significantly higher than in the control groups, particularly during the acute phase around the first 5 min, thereby confirming that *Mj*Gal plays a key role in the bacterial clearance from shrimp hemolymph and therefore, in the animal’s defense against bacterial infection. This study is the first demonstration of the biological role of a crustacean galectin in the innate immune response against infectious challenge.
